# Poc1B and Sas-6 Function Together during the Atypical Centriole Formation in *Drosophila melanogaster*

**DOI:** 10.3390/cells8080841

**Published:** 2019-08-05

**Authors:** Kyoung H. Jo, Ankit Jaiswal, Sushil Khanal, Emily L. Fishman, Alaina N. Curry, Tomer Avidor-Reiss

**Affiliations:** Department of Biological Sciences, University of Toledo, Toledo, OH 43607, USA

**Keywords:** centriole, centrosome, sperm, proximal centriole-like structure (PCL), atypical centriole, cilium, flagella, microtubule-organizing center (MTOC), Poc1B, Sas-6

## Abstract

Insects and mammals have atypical centrioles in their sperm. However, it is unclear how these atypical centrioles form. *Drosophila melanogaster* sperm has one typical centriole called the giant centriole (GC) and one atypical centriole called the proximal centriole-like structure (PCL). During early sperm development, centriole duplication factors such as Ana2 and Sas-6 are recruited to the GC base to initiate PCL formation. The centriolar protein, Poc1B, is also recruited at this initiation stage, but its precise role during PCL formation is unclear. Here, we show that Poc1B recruitment was dependent on Sas-6, that Poc1B had effects on cellular and PCL Sas-6, and that Poc1B and Sas-6 were colocalized in the PCL/centriole core. These findings suggest that Sas-6 and Poc1B interact during PCL formation. Co-overexpression of Ana2 and Sas-6 induced the formation of ectopic particles that contained endogenous Poc1 proteins and were composed of PCL-like structures. These structures were disrupted in *Poc1* mutant flies, suggesting that Poc1 proteins stabilize the PCL-like structures. Lastly, Poc1B and Sas-6 co-overexpression also induced the formation of PCL-like structures, suggesting that they can function together during the formation of the PCL. Overall, our findings suggest that Poc1B and Sas-6 function together during PCL formation.

## 1. Introduction

The centrosome is an organelle composed of two centrioles and pericentriolar material (PCM) [1]. Most animal cells have centrosomes that serve as major microtubule organizing centers (MTOCs). In dividing cells, the centrosome serves as an MTOC to assist in cell polarity and proper segregation of chromosomes. In differentiating cells, the centriole serves as a basal body to form a cilia or flagellum, which are important for cellular signaling and motility [2]. Centriole number is strictly regulated to have two centrioles per cell during mitosis by centriole duplication, and the first pair of centrioles in the zygote originates from the sperm [3,4].

Centrioles are mainly recognized by their typical barrel-shaped structure consisting of microtubules organized in a nine-fold symmetry as observed by electron microscopy. We refer to this structure as a “typical centriole” as certain cells have centrioles with a distinct structure that is atypical. Insect sperm usually have one typical centriole known in flies as the giant centriole (GC). In addition, there is an atypical centriole in the sperm of flies, beetles, and humans [5,6,7]. In flies, the atypical centriole, the proximal centriole-like structure (PCL), is made of an electron-dense material (PCL wall) surrounding a tubule-like structure (PCL tubule) in the center [6,8]. In flies, the PCL is further remodeled during sperm differentiation and become rudimentary [4]. Yet, this rudimentary PCL is functional post fertilization [9]. These observations suggest one typical and one atypical sperm centriole are paternally inherited and function as the first pair of centrioles in a fly zygote [10].

Over the last decade, much progress has been made on deciphering the duplication process of the typical centriole [11]. The pathway of new centriole assembly has been investigated based on manipulating gene expressions, protein characterization, and ultrastructure studies. In dividing cells, the new centriole first appears as a small bud next to the proximal side of the preexisting older centriole (mother centriole). At this stage, this immature centriole is known as procentriole. The procentriole formation begins with Spd-2/CEP192, Asl/CEP152, and SAK/PLK4 recruitment to the base of the mother centriole [12,13,14]. Subsequently, the core structure of the procentriole, called the cartwheel, is assembled with Ana2/STIL/Sas-5 and Sas-6/SAS6 [15,16], followed by recruitment of Sas-4/CPAP, Bld10/CEP135, Ana1/CEP295, and POC1B that are involved in centriolar microtubule assembly, elongation, and stability [17,18,19,20,21]. Finally, the PCM proteins, such as Cnn/CEP215, Asl/CEP152, and Plp/PCNT, are recruited to constitute the centrosome that serves as an MTOC [22,23,24].

Similarly, in fly sperm, the PCL first appears as a small bud next to the base of an older typical centriole, the GC, during the early stages of spermiogenesis (round spermatids) [25]. We refer to this stage as “PCL initiation.” Similar to procentriole formation, the PCL formation begins with Asl and Plk4 recruitment to the base of the GC [25,26]. During PCL initiation, Ana2, Sas-6, Poc1B, and Sas-4 are recruited to the PCL [27], followed by recruitment of other conserved centriolar proteins such as Bld10, and Ana1 [27]. Since the proteins involved in initiating the centriole and PCL formation are similar, it suggests that the initial stages of PCL and typical centriole formation are conserved [25].

The Poc1 gene family codes for conserved centriolar protein families, essential for centriole duplication, elongation, and stability [28,29]. In humans, there are two gene orthologs (POC1A and POC1B) that code for proteins that localize to the centrioles [30,31]. Disruption of human POC1A results in primordial dwarfism [32,33] and disruption of human and zebrafish POC1B results in autosomal-recessive cone-rod dystrophy [34,35]. In human cells POC1B is found in the distal lumen of the centriole [36]. In human sperm, POC1B localize to the typical centriole and atypical centriole, but in the atypical centriole it is a component of novel rods that are flanked by the uniquely splayed centriolar microtubules [7]. Immuno-EM studies in *Chlamydomonas* and *Tetrahymena* revealed that Poc1 proteins are also localized in the cartwheel, the nascent site of centriole assembly, and microtubule wall of the centriole [28,29]. In flies, the *poc1* gene codes for two splice isoforms: Poc1A, which localizes to the GC, and Poc1B, which localizes to the PCL [6]. Disruption of fly Poc1 gene expression results in infertility and early embryo development defects [6]. Altogether, Poc1 proteins are essential centriolar proteins, but their precise role during centriole formation is unclear.

Here, we report that Ana2, Sas-6, and Poc1B are colocalized at the PCL during the initial stage of PCL formation, the localization of Sas-6 and Poc1B are codependent, and Poc1B and Sas-6 are localized in the PCL/centriole core. The colocalization and codependent recruitment of Sas-6 and Poc1B to the PCL suggest that they are recruited to the PCL core at the same time. Using fluorescent microscopy, we found that Ana2 and Sas-6 co-overexpression was sufficient to form ectopic particles containing endogenous Poc1 proteins. Using serial section electron microscopy, we revealed that ectopic particles were composed of PCL-like structures and that they were disrupted in a *poc1* mutant, suggesting that Poc1 proteins are essential for the stability of PCL-like structures. Importantly, Sas-6 and Poc1B co-overexpression also induced the formation of ectopic particles composed of PCL-like structures. Overall, these findings suggest that Poc1B and Sas-6 function together during the initial stage of PCL formation.

## 2. Methods

### 2.1. Transgenic Flies


**Reference**

**Name in Text**

**Name in Ref**

**Description**
[37]Ana2GFPpUbq-Ana2-GFPAna2 cDNA tagged at the C-terminus with GFP. Expressed from an ubiquitin promoter.[37]Sas-6GFPpUbq-GFP-Sas6Sas-6 cDNA tagged at the N-terminus with GFP. Expressed from an ubiquitin promoter.[6]gPoc1BGFPPoc1A/B-GFPPoc1 gene tagged at the end of the last poc1B exon with GFP. Expressed from a *poc1* promoter.[6]Poc1BGFPUAS-Poc1B-GFPPoc1B cDNA tagged at the C-terminus with GFP. Expressed from a UAS promoter.[37]Sas-6RFPUbq-RFP-Sas-6Sas-6 cDNA tagged at the N-terminus with RFP. Expressed from an ubiquitin promoter.VDRC ID-108219Poc1RNAiPoc1-RNAiRNAi knockdown of Poc1RNAi[6]Poc1^W87X^Poc1W87XPoc1 truncation mutant[25]Sas-4GFPSas-4-GFPSas4 gene tagged at the end of the last exon with GFP. Expressed from a *sas-4* promoter.[38]Bam-Gal4P{bam-GAL4:VP16}GAL4 line expressing GAL4 from a Bam promoter.Bloomington stock #8092Poc1 defDf(3R)ED5066A chromosomal deletion that is also missing the *poc1* gene[39]
*ana2 ^719^*

*ana2*
Ana2 truncation at R175Bloomington stock #12119
*sas-4^s2214^*

*sas-4*
P-element insertion in the Sas-4 codding leading to a strong loss-of-function[13]Ana1GFPAna1-GFPAna1 gene tagged at the end of the last exon with GFP. Expressed from an *Ana1* promoter[13]gSas-6GFPgSas-6-GFPSas-6 gene tagged at the end of the last exon with GFP. Expressed from a *Sas-6* promoter[13]Ana1tdtomatoAna1-tdtomatoAna1 gene tagged at the end of the last exon with Tdtomato. Expressed from an *Ana1* promoterThis studyPoc1BΔCGFP
Poc1B cDNA lacking VLDVSIESGSDLCSCSARL DEISKLLDSIDERVRRLEGIYSL amino acid tagged at the C-terminus with GFP. Expressed from a UAS promoter.All fly stocks were cultured on standard media at 25 °C.

### 2.2. Transgenic Flies Functional Test References

**Ubq-Ana2-GFP and Ubq-GFP-Sas-6** “The homo-oligomerisation of both Sas-6 and Ana2 is required for efficient centriole assembly in flies” [40]**gPoc1B-GFP and UAS-Poc1B-GFP** “Centriole Remodeling during Spermiogenesis in Drosophila” [6]**gSas-6-GFP** “Self-assembling SAS-6 Multimer Is a Core Centriole Building Block” [41]**Ana1-tdtomato** “Drosophila Asterless the ortholog of vertebrate Cep152 is essential for centriole duplication” [13]

### 2.3. Testes Fluorescence Microscopy

Testes imaging was performed as described previously [42]. For GFP/RFP imaging, testes of adult flies were dissected in Phosphate-Buffered Saline (PBS) and imaged immediately. For antibody staining, testes of pharate adult pupae were dissected in PBS then frozen in liquid nitrogen for 5 min, fixed in 100% methanol for 1 min at 4 °C. Then, the samples were washed in PBS for 1 minute before incubation at PBST-B (PBS + 0.1% Triton + 0.5% BSA) for 15 min at room temperature. Then it was incubated in the primary antibodies in PBST-B for 12 hours at 4 °C and washed 3 times with PBST (PBS + 0.1% Triton) for 5 minutes each, followed by incubation in secondary antibodies for 90 min at room temperature. Lastly, the sample was washed 3 times with PBST (PBS + 0.1% Triton) then PBS for 5 min each. For quantification, pictures were taken in the photon counting mode using a 63× objective with a zoom of 6, pin hole of 1.0, resolution of 512 × 512 pixels, 488 nm laser power of 1% of 20%, and using a measuring box of 1 µm by 2.5 µm that was placed on top of the centriole and the PCL. Images were taken by SP8 scanning confocal microscope (Leica Biosystems, Wetzlar, Germany), Z stacks at room temperature, and analyzed using Leica software. For photon counting, about 1–2 days old male flies were analyzed. For each fly, a pair of testes was dissected and photons were counted from round spermatid centrioles. Maximal projection images were then modified using Adobe Photoshop (**Adobe** Systems Inc., San Jose, CA, USA) and annotated using Adobe Illustrator. We used photoshop to crop the relevant information from an image. We increased the overall signal when it was dim to help readers to see it. All changes were made to the whole image in accordance with the standard guidelines.

### 2.4. Antibodies

Anti-N-Poc1 antibodies were described in [6]. Anti-N-Poc1 was made in rabbits Anti-N-Poc1, designed to detect both Poc1 isoforms, and recognizes amino acids 6 to 20 (RDPALERHFTGHSGG). The following antibodies were used for immunofluorescence at indicated concentrations: Primary antibodies for immunofluorescence: Ana2 and Sas-6 antibodies 1:50 (Gift from Jordan Raff); Anti-Poc1 N-terminus (3218) at 1:100; Gamma tubulin antibodies (GTU88) at 1:100. Secondary antibodies for immunofluorescence: 1:400 Cy™5 AffiniPurified Goat Anti-Rabbit IgG (H+L), 1:400 Rhodamine Red-X-AffiniPure Goat Anti-Rabbit. IgG (H+L)., 1:200 (Jackson ImmunoResearch Laboratory, Philadelphia, PA, USA) at 1:1,500 in 5% BSA.

### 2.5. Tissue Processing and Transmission Electron Microscopy

For TEM analysis of PCL-like structures and centriole ultrastructure, the testes and seminal vesicles of flies were dissected and immediately fixed in 2.5% Glutaraldehyde in 0.1 M Cacodylate buffer (PH.7.2) for 2 h at 4 °C. The fixed tissue was rinsed 3 times with Cacodylate buffer for 15 minutes each. Then the tissue was en bloc stained in 1% OsO_4_ in 0.1 M Cacodylate buffer at room temperature for 30 minutes. After, the tissue was rinsed 3 times with double distilled water for 10 minutes each. Then the tissue was further stained with 0.5% Uranyl acetate in water for 12 hours at 4 °C. The tissue was dehydrated using 50%, 70%, 90%, and 100% acetone in water for 15 minutes each. Lastly, the tissue was infiltrated and embedded in EMBed 812 resin at room temperature and then polymerized at 60 °C for 12 h. The ultrathin sectioning (70 nm) was performed using ultramicrotome (Leica EM UC6, Leica, Wetzal, Germany), and sections were poststained with 6% Uranyl Acetate (in 1:1 70% ethanol and 100% methanol), and Reynolds Lead citrate (3%–4%) (in preboiled ddH2O). Then, the sections were imaged using TEM (JEOL 1400-plus, JEOL, Peabody, MA, USA) operating at 80 kV. When measuring the diameter of PCL-like structures, each structure was measured in 3 different angles and averaged using ImageJ software version 1.51w (NIH, Bethesda, MD, USA).

### 2.6. Super-Resolution Microscopy

Direct stochastic optical reconstruction microscopy (dSTORM) data were acquired using a Leica DMI8-S multi-color TIRF high power microscope (Germany) equipped with a 160x objective name. Images were collected with a Hamamatsu flash 4D camera (Japan) with a 6.5 µm pixel and a 0.4× c-mount to deliver a 100 nm pixel with a final resolution of 20 nm. This objective has a 800 nm depth of field, which delivers a Z resolution of at least 200 nm. The fluorophores were excited with a 150 mW 633 nm laser, which delivered 75 mW to the objective. Images were depleted and then acquired with a laser power of 100%. The exposure time was 10 ms. A quadruple-pass filter was used to collect fluorescence from samples labeled with Alexa 647 conjugated secondary antibodies. The data were processed using Leica GSD tools with a 2.2 photon to electron conversion factor (analog to digital conversion factor) and a 100 nm pixel size. The centriole and the PCL diameters were determined using ImageJ software version 1.51w (NIH). The distance between two ends of the base line of the intensity graph was determined for each measurement, and the average distance for three different angles was calculated for each centriole/PCL diameter. Identical measurements were performed for 26 centrioles expressing Sas-6GFP, 38 PCLs expressing gPoc1BGFP, and 43 PCLs expressing Ana1GFP. The graph and the statistical analysis (two tailed student T-test) were performed using Microsoft Excel and Adobe Illustrator (Adobe, San Jose, CA, USA).

### 2.7. Statistical Methods

Experiments were repeated at least three times, and statistical analyses (SEM+/−) were done with excel. A two-tailed, unpaired Student’s T-test was used. Data are presented as average ± standard deviation as calculated by Microsoft Excel (USA).

### 2.8. Reagent and Data Availability

10x PBS recipe: https://www.protocolsonline.com/recipes/phosphate-buffered-saline-pbs/. Reagents for PBST: Triton X-100 at Sigma-Aldrich, St. Louis, MO, USA (CAS# 9002-93-1), BSA (albumin fraction V) at Chem-Impex-International, Wood Dale, IL, USA (CAS# 9048-46-8), Ana2, and Sas-6 antibodies were kindly provided from Jordan Raff laboratory. Gamma tubulin antibodies (GTU88) at Sigma-Aldrich (Catalog No. T6557) Cy™5 AffiniPure Goat Anti-Rabbit IgG (H+L) at Jackson Immuno Research Laboratory, Philadelphia, PA, USA (Code: 711-175-152), Rhodamine Red-X-AffiniPure Goat Anti-Rabbit IgG (H+L) at Jackson Immuno Research (Code: 111-295-144), Alexa Fluor 647 at Jackson Immuno Research (Code 711-606-152), and Poc1 N-terminus antibody was made within our lab and is available upon request. Glutaraldehyde at Electron Microscopy Sciences (CAS #111-30-8). Cacodylate buffer was kindly provided by Dr. William Gunning at the University of Toledo. EMBed 812 resin at Electron Microscopy Sciences (CAS #13940). Reynolds Lead citrate at Electron Microscopy Sciences (CAS #10099-74-8). ImageJ software is available at https://imagej.nih.gov/ij/.

## 3. Results

### 3.1. Poc1B Is Recruited during the Initial Stage of the PCL Formation

**Figure 1:**
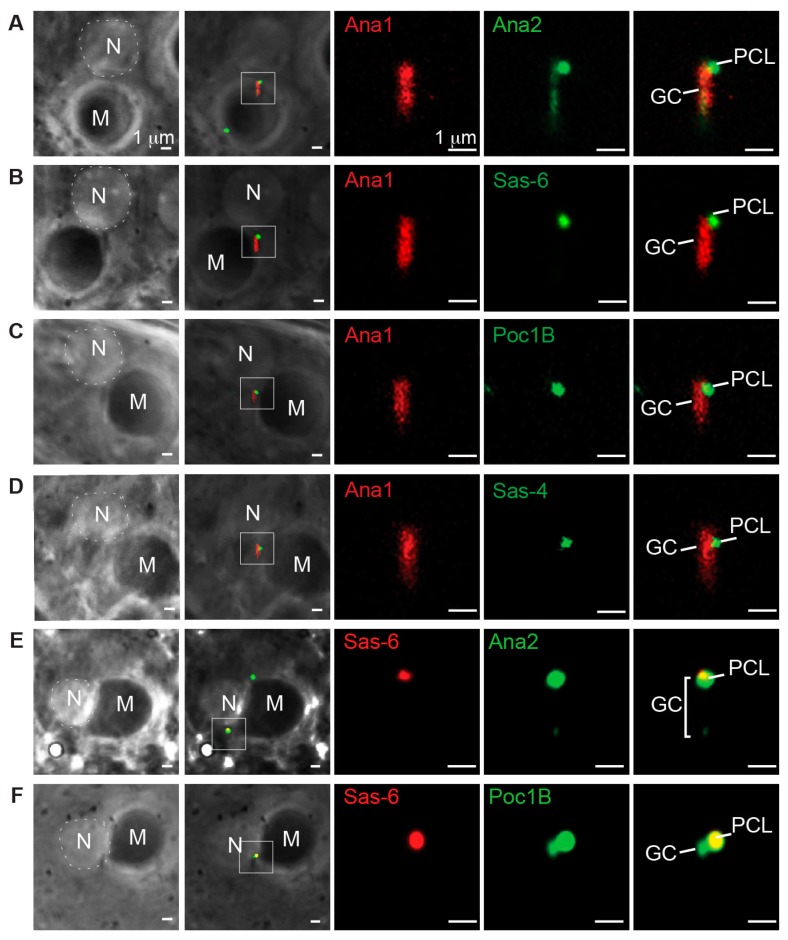
Poc1B is recruited during the initial stage of proximal centriole-like structure PCL formation. (**A**–**F**) Proteins localized at the centrioles in round spermatids. Low magnification images of phase contrast on the left panel showing the round shape of nucleus (N) and Mitochondria derivatives (M) relative to the centrioles. High magnification images of centrioles on the right panels. The magnified region is marked with the white box. Phase contrast images were used to determine the same stage of round spermatids for each protein. All images were obtained by live cell imaging. (**A**) Ana2GFP and Ana1tdtomato label the giant centriole (GC), but only Ana2GFP is enriched at the PCL. (**B**) Sas6GFP and Ana1tdtomato label the GC, but only Sas6GFP is enriched at the PCL. (**C**) Ana1tdtomato label the GC, but only gPoc1BGFP is enriched at the PCL. (**D**) Ana1tdtomato labels the GC, but only Sas-4GFP labels the PCL. (**E**) Ana2GFP and Sas6RFP are colocalized at the PCL. (**F**) Poc1BGFP and Sas6RFP are colocalized at the PCL.

Upon meiosis exit, the PCL first appears as a small bud on the side of the GC base [25]. To determine the timing of Poc1B localization to the PCL, we selected the earliest stage in spermiogenesis (round spermatid), when PCL is first labeled by Ana2, Sas-6, and Sas-4 tagged with GFP. Using live cell imaging, spermatid stages were identified based on the shape of the nucleus and mitochondria derivatives using phase microscopy. In round spermatids both the nucleus and mitochondria derivatives were round with a similar size (Figure 1A–C). In later stages, the nucleus and mitochondria derivatives elongated [43]. We used Ana1 tagged with tdTomato to label the GC because Ana1 is only localized at the GC in early spermiogenesis. As expected, Ana1 was only localized to the GC and Ana2, Sas-6, Poc1B, and Sas-4 were localized to the PCL in early round spermatids (Figure 1A–D). However, Ana2 was also observed at a low level along the GC (Figure 1A). The localization to the GC is most likely due to overexpression by a strong ubiquitin promoter. Since Poc1B and Sas-4 are expressed by an endogenous promoter, they were only observed in the PCL (Figure 1C,D). Importantly, Poc1B and Ana2 colocalized with Sas-6 at the PCL in round spermatids (Figure 1E,F). Altogether, these findings indicate that Poc1B is recruited during PCL initiation in round spermatids and may function together with Ana2, Sas-6, or Sas-4 to begin the PCL formation.

### 3.2. Poc1B Is Downstream to Ana-2 or Sas-6 during PCL Initiation

**Figure 2:**
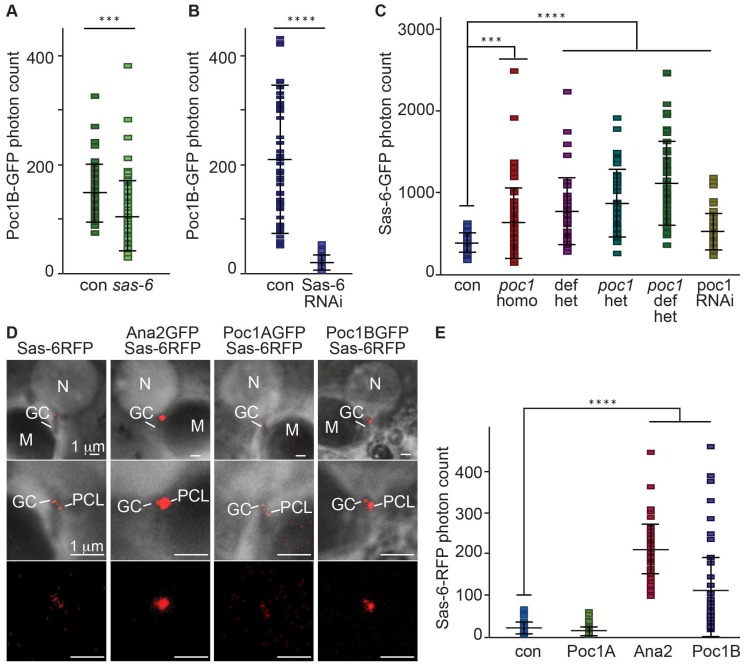
Poc1B is downstream to Sas-6 during PCL initiation. (**A**,**B**) Localization of gPoc1BGFP to the PCL was slightly reduced in *sas-6* heterozygote mutant background (N = 63) compared to the control (con, (N = 46) (A) and much more reduced in *sas-6* RNAi background (N = 72) (B) compared to control (con, N = 52). gPoc1BGFP was expressed using endogenous promoter. (**C**) Sas6GFP localization to the PCL was significantly increased in *poc1*^W87X^ homozygote mutant (N = 55), Poc1 deficiency (Df(3R)ED5066) transheterozygote (N = 43), *poc1*^W87X^ heterozygote (N = 42), *poc1* deficiency with *poc1*^W87X^ (N = 55), and Poc1 RNAi(P{KK102200}VIE-260B) (N = 58) background compared to the control (con, N= 44). Sas6GFP was expressed using ubiquitin promoter. (**D**,**E**) Sas6RFP localization to the PCL was significantly increased in Ana2GFP (N = 44) and Poc1BGFP (N = 42) overexpressing flies but not in Poc1AGFP overexpressing flies (N = 70) or the control (con, N = 61) (D). Quantification of Figure 2D (**E**). Sas6RFP was expressed using ubiquitin promoter. All images and quantification data were obtained by live cell imaging. Con, Control; Het, Heterozygote; Homo, Homozygote; OE, Overexpression. ***p < 0.001. ****p < 0.0001.

To study the function of Poc1B during PCL initiation, we quantified the localization of proteins recruited during PCL initiation in different mutant backgrounds. First, we quantified Poc1B localization to the PCL in Ana-2, Sas-6, and Sas-4 heterozygote mutants, as well as the Sas-6 RNAi condition. The RNAi was strongly expressed in sperm cells using Bam-GAL4-VP16 [44,45]. We found that the Poc1B localization to the PCL was slightly reduced in *sas-6^c02901^ heterozygote* (*p* value: 0.0001), and significantly reduced in Sas-6 RNAi (*p* <0.0001) (Figure 2A,B) and in *ana2^719^ heterozygote* mutant flies (*p* = 0.0118) (Figure S1). However, Poc1B localization to the PCL was not affected in *sas-4^s2214^ heterozygote* mutant flies (*p* = 0.5041) (Figure S1). These findings suggest that Poc1B localization to the PCL is dependent on Ana-2 or Sas-6, but not on Sas-4.

Next, we tested whether Poc1 proteins affect Ana2, Sas-6, and Sas-4 localization to the PCL. We quantified their localizations to the PCL in the *poc1* loss-of-function allele (homozygote) and the Poc1RNAi condition (Poc1A and Poc1B knockdown). We found that Ana2 localization is not affected (*p* = 0.3117). This suggests that Ana2 is recruited upstream of Poc1 and is consistent with our finding that Poc1B localization to the PCL is dependent on Ana-2.

We also found that Sas-4 localization to the PCL is significantly reduced in *poc1* loss-of-function alleles (*p* < 0.0001) (Figure S2). This suggests that Sas-4 localization is dependent on Poc1 proteins and is consistent with our finding that Poc1B localization to the PCL is independent of Sas-4.

Unexpectedly, we found that Sas-6 localization is increased in *poc1* loss-of-function allele (homo-, hetero-, hemi-, and trans-heterozygote) (*p* = 0.0003, <0.0001, <0.0001) and Poc1RNAi condition (*p* = 0.0001) (Figure 2C). This suggests that Poc1 proteins are not essential for Sas-6 recruitment, agreeing with our finding that Poc1B localization to the PCL is downstream to Sas-6 and has some inhibitory effect on Sas-6 level in the PCL. The unexpected inhibitory effect may be part of a feedback regulatory mechanism and is beyond the scope of this study.

### 3.3. Poc1B Overexpression Induces Increase Cellular and PCL Sas-6 Level

In typical centriole formation, Ana2 is upstream to Sas-6. Ana2 directly interacts and promotes Sas-6 recruitment to the procentriole cartwheel in flies and humans [11,46], Therefore, we quantified Sas-6 localization in Ana2 overexpressing flies. As expected from the interaction between the proteins, Sas-6 localization to the PCL was significantly increased in Ana2 overexpressing flies (*p* < 0.0001) (Figure 2D,E). This suggests that, like in the typical centriole, Ana2 interacts with Sas-6 in the PCL as well.

We found that PCL Poc1B is downstream to Sas-6, but how far downstream is unknown. To test if Poc1 is immediately downstream to Sas-6, we tested if the two proteins interact genetically. For this aim, we quantified Sas-6 localization in Poc1A and Poc1B overexpressing flies. We found that Sas-6 localization is not affected in Poc1A overexpressing flies. However, the Sas-6 localization was significantly increased in Poc1B overexpressing flies (*p* < 0.0001) (Figure 2D,E). This suggests that the Poc1B isoform interacts with Sas-6 to affect its localization to the PCL.

To get insight into how Poc1B overexpression led to an increase of Sas-6 in the PCL, we determined the level of Sas-6-GFP in the testes using Western blot. We found that Sas-6-GFP protein expression is significantly increased in Poc1B overexpressing flies compared to control flies. This suggests that Poc1B can increase cellular Sas-6 in vivo (*p* < 0.05) (Figure S3). We think it is likely that this increase is mediated by protein stability and not by an increase of mRNA level or an increase of translation, because the Sas-6 in this experiment was expressed by a plasmid (Sas-6-GFP) that lacks Sas-6’s native regulatory sequences. Altogether, our findings suggest that Poc1B interacts with Sas-6 to regulate its level in the cell and its localization in the PCL.

### 3.4. Poc1B and Sas-6 Are Localized in the PCL Core

**Figure 3:**
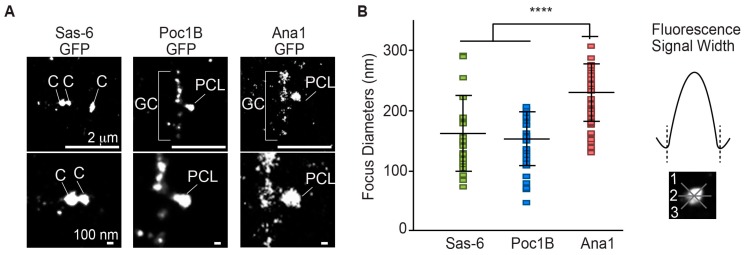
Poc1B and Sas-6 localize in the PCL core. (**A**) Maximum intensity projection of direct stochastic optical reconstruction microscopy (dSTORM) super-resolution micrographs of sperm centrioles containing gSas-6-GFP, gPoc1B-GFP, and Ana1-GFP. Sas-6 diameters were obtained from the spermatocyte centrioles. Poc1B and Ana1 were obtained from elongating spermatids PCL. (**B**) The diameter quantification of dSTORM micrographs of gSas-6-GFP (N = 26), gPoc1B-GFP (N = 38), and Ana1-GFP (N = 43). Sas-6 and Poc1B had similar diameters that were significantly smaller than Ana1. Each measurement was measured 3 times as depicted on the right. ****p < 0.0001.

Next, we investigated Poc1B localization within the PCL. Previously, we reported using SIM-3D that Poc1B C-terminus tagged with GFP labeled at a focus of ~280 nm in diameter and Ana1 C-terminus tagged with GFP (Ana1-GFP) labeled at a focus of ~370 nm in diameter. We suggested that Poc1B is localized closer to the PCL core, like Sas-6 compared to Ana1 [6]. To determine more precisely the localization of Poc1B in the PCL, we compared Poc1B and Sas-6 localization using direct stochastic optical reconstruction microscopy (dSTORM) (Figure 3A). We used the GFP antibody against Poc1B-GFP, Sas-6-GFP, and Ana1-GFP (GFP tagged on the C-terminus) to measure the diameter of their fluorophore (Figure 3B). Since it is known that the Sas-6 localization to the centrioles is reduced in spermatids, we measured the diameter of Sas-6 fluorophore in spermatocyte centrioles. We found that Sas-6 has the diameter of 150 ± 58 nm. The diameters of Poc1B and Ana1-GFP fluorophores in the PCL were 141 ± 40 and 213 ± 44 nm, respectively (Figure 3B). The new, narrower measurements (of Ana1-GFP ~210 versus ~370 nm; Poc1B-GFP ~140 versus 280nm) were probably due to improved resolution of STORM over SIM. These results showed that Poc1B and Sas-6 foci had similar diameter (~140 versus ~150) and were significantly smaller than the Ana1 (*p* < 0.0001), suggesting that Poc1B and Sas-6 are localized within the centriole/PCL core.

### 3.5. Ana2 and Sas-6 Overexpression Can Induce PCL-Like Structure That Contains Poc1

Overexpression of early centriole duplication factors have the unique ability to induce procentriole formation. In human cells, overexpression of the early centriole duplication factors Plk4, Ana2, and Sas-6 induce the formation of an abnormally large number of procentrioles around the preexisting centriole in human cells [47,48,49]. In contrast, the overexpression of the centriole duplication factors Plk4, Ana2, and Sas-6 in fly sperm induce the formation of large ectopic particles, which contain early intermediates in centriole formation [46]. These particles are found freely suspended in the cytoplasm or connected to the two centrioles in primary spermatocytes. The early intermediates in the ectopic particles resemble the PCL ultrastructure: Containing a core that is made of an extended tubule that is surrounded by an electron-dense material similar to the “PCL wall”. We therefore refer to them as “PCL-like structures” [6] (Figure S4).

**Figure 4:**
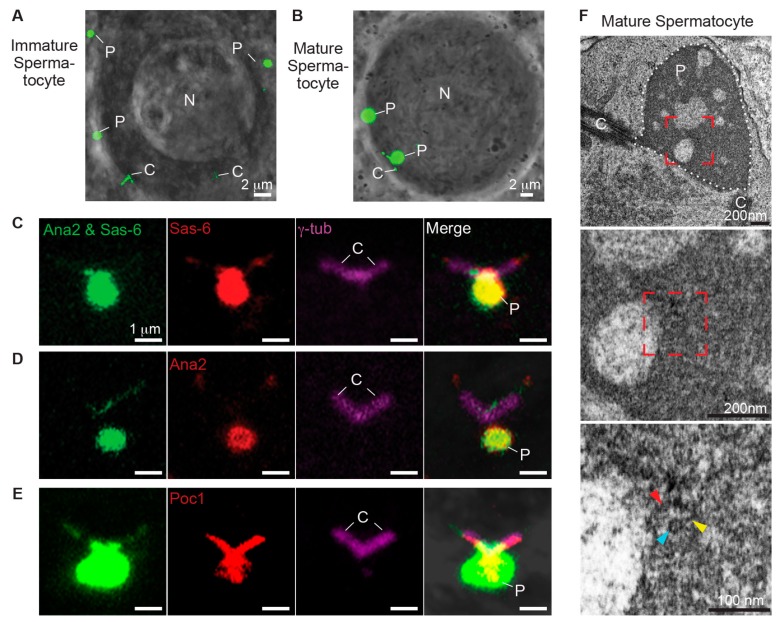
Ectopic particles contain Poc1B. (**A**,**B**) Co-overexpression of Ana2GFP and Sas-6GFP induced the formation of ectopic particles. Immature spermatocyte contained two centrioles (C) and ectopic particles (P) in the cytoplasm (A). Mature spermatocyte contained ectopic particles connected to the two centrioles and in the cytoplasm (B). Images were obtained by live cell imaging. (**C–E**) Ectopic particles were attached to the γ-tubulin labeled centrioles. Sas-6 antibodies labeled the particle and the centrioles’ tip (**C**), Ana2 antibodies labeled the particle and centrioles’ tip (**D**), and Poc1 antibodies labeled the particle and the centrioles (**E**). (**F**) Ectopic particles were composed of PCL-like structures in mature spermatocytes. Low electron microscopy magnification (upper row) showed dense PCL-like structures attached to the two centrioles (red dotted box). The electron dense area is outlined with white dotted lines. Intermediate magnification (middle row) showed PCL-like structures embedded in an electron-dense area. High magnification (lower row) showed that PCL-like structures consisting of tubule-like structures (blue arrowhead), spokes (yellow arrowhead), and electron-dense material (red arrowhead) in mature spermatocytes. C, Centrioles; P, Particles.

Both the formation of the ectopic particles and PCL require Plk4, Ana2, and Sas-6, and their ultrastructure is similar in that they both resemble intermediate structure during procentriole assembly. Therefore, we tested the function of Poc1B and Sas-6 interaction during ectopic particle formation. We found that Ana2 and Sas-6 (Ana2–Sas-6) co-overexpression without Plk4 induced formation of the ectopic particles in primary spermatocytes and some particles were attached to the centrioles only in mature primary spermatocytes (Figure 4A,B). This finding suggests that Ana2 and Sas-6 are sufficient to induce ectopic particle.

To test whether Poc1 proteins are components of the ectopic particles in Ana2–Sas-6 co-overexpressing flies, we used antibodies against Ana2, Sas-6, and Poc1 (anti-Poc1 recognizes both Poc1A and Poc1B isoforms). Also, an antibody against γ-tubulin was used to label the centrioles. As expected, Sas-6 and Ana2 antibodies labeled the particles (Figure 4C,D). We also found that Poc1 antibodies labeled the particles (Figure 4E). These findings indicate that endogenous Poc1 proteins are components of ectopic particles, as observed in the PCL.

Next, we evaluated the ultrastructure of the ectopic particles using serial section transmission electron microscopy. Consistent with findings by Stevens et al., we found electron-dense regions connected to the centrioles that are composed of structures resembling the PCL ultrastructure (Figure 4F). These PCL-like structures consisted of electron-dense materials with the diameters of 119 ± 15 nm (N = 122) and tubules with the diameters of 20 ± 2 nm (N = 17) (Figure S5). Altogether, our findings suggest that Ana2 and Sas-6 co-overexpression is sufficient to induce ectopic particles composed of Poc1 proteins and PCL-like structures.

### 3.6. Poc1 Proteins Are Essential for Proper Formation of PCL-Like Structure

**Figure 5:**
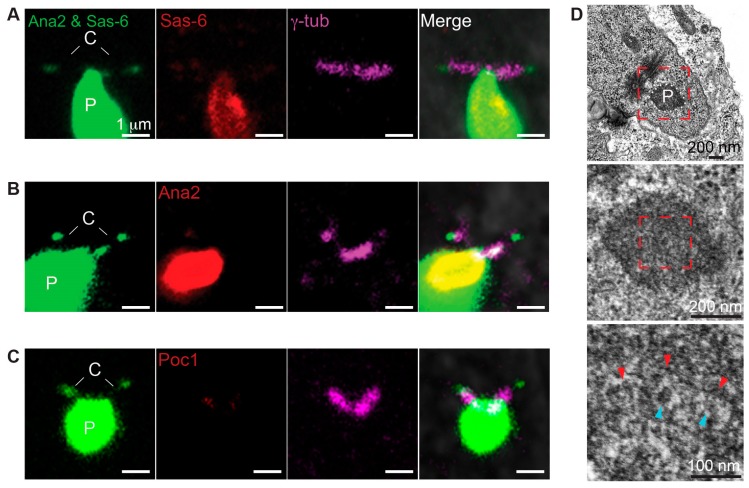
Poc1 is essential for proper formation of PCL-like structure. (**A**–**C**) Sas6/Ana2 Particles in *poc1*^W87X^ homozygote were attached to the γ-tubulin labeled centrioles. Sas-6 antibodies labeled the particle (**A**), Ana2 antibodies labeled the particle (**B**), and Poc1 antibodies weakly labeled the centrioles and did not label the particle (**C**). (**D**) Sas6/Ana2 particles in *poc1*^W87X^ homozygote had deformed PCL-like structures in mature spermatocyte. Low electron microscopy magnification (upper row) showed PCL-like structures near the electron dense area, outlined with white dotted lines. Intermediate and high magnification showed that PCL-like structures were composed of fragmented tubule-like structures (blue arrowhead) and electron-dense material (red arrowhead) in mature spermatocyte (middle and lower row).

Since endogenous Poc1 proteins are components of the ectopic particles, we co-overexpressed Ana2 and Sas-6 in *poc1^W87X^* loss-of-function mutant flies to study the role of Poc1 proteins. We found that the particles were present in primary spermatocytes in *poc1^W87X^* mutant flies (Figure 5A–C). As expected, Ana2 and Sas-6 antibodies labeled the particles while Poc1 antibody did not (Figure 5A–C). These observations suggest that Poc1 proteins are dispensable for inducing ectopic particles.

Previously, we reported that PCL can be observed by fluorescent microscopy during PCL initiation, but PCL ultrastructure was disrupted in *poc1^W87X^* loss-of-function mutant flies [6]. Therefore, we tested the structural role of Poc1 proteins in formation of the PCL-like structures using TEM. We observed the electron-dense regions connected to the centrioles composed of disrupted PCL-like structures (Figure 5D). The tubule-like structures were difficult to identify, and surrounding electron dense materials were fragmented. These observations suggest that the Poc1 proteins are essential for forming stable PCL-like structures, which is consistent with the role of Poc1 in PCL formation.

### 3.7. Poc1B and Sas-6 Co-Overexpression Is Sufficient to Induce PCL-Like Structures

**Figure 6:**
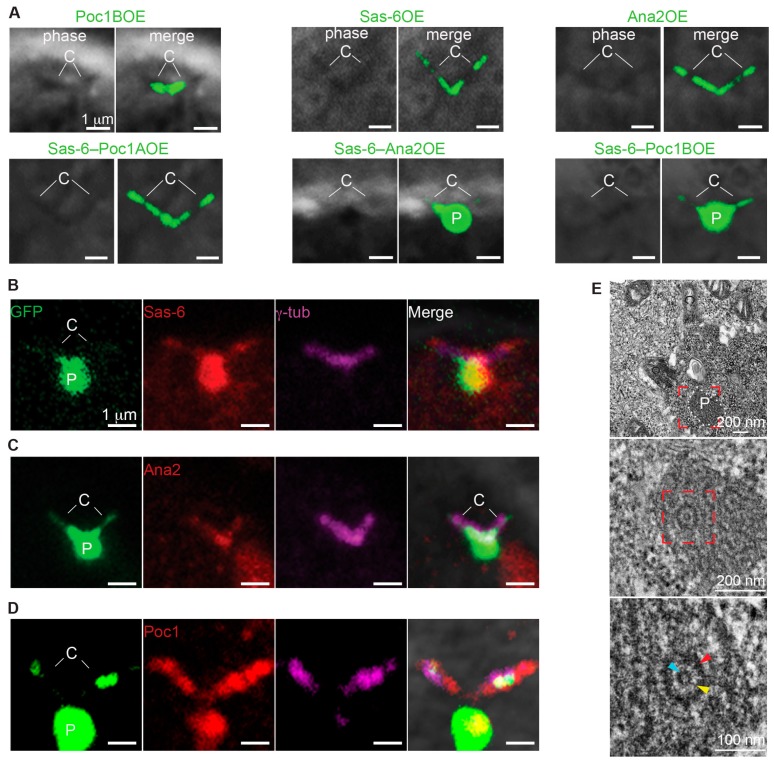
Co-overexpression of Poc1B and Sas-6 is sufficient to induce formation of PCL-like structures. (**A**) Co-overexpression of either Sas-6GFP–Ana2GFP or Sas-6GFP–Poc1BGFP induced the particles in primary spermatocyte. No particles were observed in co-overexpression of Sas-6GFP– Poc1AGFP or in overexpression of any of the tested proteins alone. (**B**–**D**) Sas6/Poc1B Particles were attached to the γ-tubulin labeled centrioles, Sas-6 antibodies labeled the particles and the centrioles tip (**B**), Ana2 antibodies labeled the centrioles (C), and Poc1 antibodies labeled the particles and centrioles (**D**). (**E**) Sas6/Poc1B Particles were composed of PCL-like structures in mature spermatocyte.

Since Poc1 proteins are essential for forming stable PCL-like structures, we tested whether overexpressing Poc1A or Poc1B could also induce PCL-like structures. We observed that in addition to Ana2–Sas-6 (80.8% of the spermatocytes), Sas-6–Poc1B co-overexpression could also induce the formation of particles in primary spermatocytes (82.8% of the spermatocytes). In contrast, Ana2–Poc1B (9.9% of the spermatocytes) or Sas-6–Poc1A (0% of the spermatocytes) co-overexpression did not induce the particle formation (Figure 6A and graph Figure S6). This strongly suggests that Sas-6 and Poc1B isoform interact to induce the formation of particles in primary spermatocytes.

Next, we determined the protein composition of the centriole-attached particles using Ana2, Sas-6, and Poc1 antibodies in Sas-6–Poc1B co-overexpressed flies. As expected, Sas-6 and Poc1 antibodies labeled the particles. Surprisingly, the Ana2 antibody labeled only the centrioles’ base but not the particles (Figure 6B–D). This demonstrates that Sas-6 and Poc1B co-overexpression is sufficient to induce the formation of the particles not composed of extra Ana2.

Lastly, we studied the ultrastructure of the Sas-6/Poc1B induced ectopic particles and found that these particles were composed of PCL-like structures as well (Figure 6E). These PCL-like structures consisted of electron-dense materials with the diameters of 118 ± 16 nm (N = 90) and tubule with the diameters of 24 ± 2 nm (N = 17) (Figure S5A). We found that the electron-dense material had similar diameters, but the tubule diameters were significantly wider in Sas-6–Poc1B co- overexpressing flies compared to Sas-6–Ana2 co-overexpressing flies (20 ± 2 nm) Figure S5A). These findings suggest that Poc1B overexpression is involved in the formation of tubule-like structures and Sas-6–Poc1B co-overexpression is sufficient to induce PCL-like structures without recruiting detectable amount of Ana2 to their structure. Altogether, these findings strongly suggest that Sas-6 and Poc1B interaction is important during the initial stages of PCL formation.

### 3.8. The Coiled Coil Domain of Poc1B Is Essential to Induce Ectopic Particles

**Figure 7:**
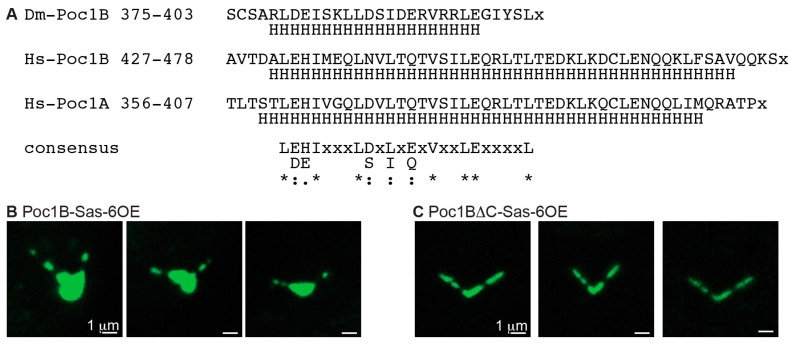
The coiled coil domain of Poc1B is essential to induce ectopic particles. (**A**) The coiled coil region of fly Poc1B is conserved in human Poc1A and Poc1B. The C-terminus of fly (Dm) Poc1B (amino acid residues 375–403), Human (Hs) Poc1B (amino acid residues 427–478), and Human Poc1A (amino acid residues 356–407) has a conserve coiled coil region. This region has a conserved amino acid sequence: LEHIxxxLDxLxExVxxLExxxxL. H; amino acid residue predicted to be part of a coiled coil domain, *; identical amino acid residue, “:”; conserved amino acid residue, “.”; weakly similar amino acid residue, x; stop codon. (**B**,**C**) While co-overexpression of Sas-6GFP–Poc1BGFP induced ectopic particles in primary spermatocyte (**B**), no particles were observed in co-overexpression of Sas-6GFP–Poc1BΔC-GFP (C).

Next, we studied the similarity between fly and human Poc1 proteins by using protein structure prediction software. We compared the protein structure of fly Poc1A and Poc1B splice isoforms with human Poc1A and Poc1B. Protein structure prediction showed that all four proteins have seven WD repeats in the N-terminus and an unstructured region in the middle [6]. However, only human POC1A, human POC1B, and *Drosophila* Poc1B were predicted to have a conserved coiled coil domain on the C-terminus (Figure 7A). *Drosophila* Poc1A appeared not to have this coiled coil domain. Since Poc1B and not Poc1A overexpression with Sas-6 induced the formation of particles composed of PCL-like structures, this finding suggest that the conserved coiled coil domain is important for functional interaction between Poc1B and Sas-6 during centriole formation in flies and humans.

To test the significance of the C-terminal coiled coil domain, we created Poc1B truncation construct tagged with GFP without the C-terminus in flies (Poc1ΔC-GFP). Then, we tested the ability of Poc1ΔC-GFP to form ectopic particles. We found that co-overexpression of Sas-6 and Poc1BΔC-GFP failed to form ectopic particles (Figure 7C). This supports the idea that the conserved coiled coil domain on the C-terminus is needed for Sas-6 interaction.

## 4. Discussion

We concluded that Poc1B and Sas-6 interaction is important for the PCL formation in *Drosophila melanogaster*. In support of that we found:

1. Poc1B and Sas-6 were recruited during the initial stage of PCL formation.
2. Poc1B and Sas-6 were localized in the PCL core.
3. Poc1B recruitment was dependent on Sas-6.
4. Poc1 mutation increased Sas-6 localization to the PCL.
5. Poc1B overexpression increased Sas-6 in the testes and PCL.
6. Overexpression of Ana2 and Sas-6 induced the formation of atypical particles in PCL-like structures that include Poc1 proteins.
7. Poc1 proteins were essential for forming stable PCL-like structures.
8. Overexpression of Poc1B and Sas-6 induced the formation of PCL-like structures.
9. The C-terminal coiled coil of Poc1B was essential for formation of ectopic practical.

### 4.1. PCL Could Be Homologous to the Intermediate Structure during Pro-Centriole Formation

The typical procentriole and the PCL share several characteristics: Plk4, Ana2, and Sas-6 are required to initiate both the procentriole and the PCL formation [12,16,19,50], and the ultrastructure of procentriole without the microtubules and the PCL are both made of an electron dense wall with a tubule-like structure in the center [51]. Therefore, we hypothesized that the PCL is an intermediate structure that is arrested before it develops into a typical procentriole in the fly sperm.

Based on review of the literature on procentriole formation pathway and the new data we present here, we propose the following mechanism for PCL formation: First, the PCL formation begins with Asl and Plk4 recruitment to the formation site [25,26]. Second, Plk4 activates Ana2 to be recruited during the PCL initiation (Figure 2) [52]. Third, the phosphorylated Ana2 binds with Sas-6 to begin the cartwheel assembly during the PCL initiation [52]. Fourth, Poc1B is recruited to interact with Sas-6 to form the PCL tubule. Lastly, the Sas-4 and Ana1 are recruited to further develop the PCL [25,53].

The investigation of centriole assembly during the past several years has resulted in remarkable findings. However, the precise functional and structural roles of the early centriolar proteins are still unclear. Here, we demonstrated that Poc1B interacted with Sas-6 during PCL initiation in the flies. Since Poc1B is a conserved centriolar protein, we speculate that the Poc1B and Sas-6 interaction occurs not only in the flies but also in other animals during typical centriole formation. Further studies are necessary to resolve the precise role of Poc1 proteins during cartwheel or procentriole formation.

One way to explain the arrested nature of the PCL is via the developmental evolutionary term Neoteny [54]. Neoteny is a decrease in the rate of development or a maturation arrest at an early stage. In the centriole of human cells, POC1B is recruited near the end of procentriole formation [21] via human CEP295. In the fly PCL, the order of recruitment seems to be reversed; the fly Poc1B is essential for the recruitment of the fly CEP295 [25]. Together, these studies suggest that Poc1B gains a new essential early function in the centriole formation pathway in flies that is not observed in human centrioles. This new essential function may allow the cartwheel to be a stable structure and become the PCL, instead of being an intermediate structure that normally continues to develop into a stable centriole.

### 4.2. Poc1B May Be a Component of the Cartwheel Inner Density

Analysis of the procentriole assembly in in vivo and in vitro experiments identified the centriolar protein Sas-6 as one of the key components of the cartwheel found in the center of centriole base [55]. The cartwheel assembly is the initial step in procentriole formation. Recent studies have suggested that there is an unknown electron-dense entity, called the cartwheel inner density (CID) found within the cartwheel hub [56]. However, the function and composition of CID are unknown.

Interestingly, a structure resembling CID has been identified in the PCL [6]. The early spermatid PCL is composed of electron-dense entities: The “PCL tubule” found in the center and the “PCL wall” surrounding it (Figure S4). At the end of sperm development (in spermatozoa), the PCL wall is lost, Sas-6 is reduced, and the PCL tubule is attenuated. At this stage Poc1B is the only known protein retained in the spermatozoa PCL. This suggests that Poc1B is a component of the attenuated PCL tubule. Consistent with these data, the tubule diameters of the PCL-like structures (~24 nm) were similar to PCL tubule diameter when Poc1B was co-overexpressed with Sas-6 (24 ± 2 nm), in contrast to when Ana2 was co-overexpressed with Sas-6 (20 ± 2 nm) (Figure S4A) [6]. Therefore, we speculate that Poc1B and Sas-6 function together during PCL initiation to form the PCL tubule and cartwheel hub, and then the cartwheel and its hub composed of Sas-6 is lost, resulting in an attenuated PCL tubule composed of Poc1B that represents the CID in fly sperm centriole. Since Poc1B is a conserved centriolar protein in flies and humans, we speculate that Poc1B could be one of the CID components found in typical centrioles in humans.

### 4.3. Poc1B May Have Multiple Functions in Centriole Assembly

Poc1B was reported to be recruited after CEP295 near the end of centriole formation [21], and it was reported to be located in the distal centriole lumen [36] of human cells. More recently, Poc1B was suggested to be a component of the link connecting the A tubule to the adjacent C tubule of triplet microtubule blades in *Chlamydomonas* [57]. A similar function for Poc1 as a linker between microtubules was proposed in *Tetrahymena* [58,59]. Considering that the PCL does not have microtubules, then the role of Poc1B in the PCL is independent of POC1’s role in stabilizing microtubules by linking them. Also, considering that the PCL does not have a distal lumen, as it mostly resembles the cartwheel, then the role of Poc1B in the PCL is independent of POC1’s role in the distal lumen. Altogether, it appears that Poc1B has multiple roles in the centriole, including functioning in early, middle, and late stages of the centriole formation pathway.

## Supplementary Materials

The following are available online at https://www.mdpi.com/2073-4409/8/8/841/s1, Figure S1: Poc1B recruitment is reduced in ana2 but not in sas-4 heterozygote mutants, Figure S2: Ana2 is not affected and Sas-4 is reduced in poc1 mutant and knockdown, Figure S3: Sas-6-GFP expression increases in Poc1B overexpressing flies, Figure S4: Ectopic particles contain PCL-like structures, Figure S5: PCL-like structure outer and tubule diameters, Figure S6: Sas-6-Ana2 and Sas-6-Poc1B specifically induce the formation of centriole-attached particles.

## References

[1] Bornens M. (2012). The centrosome in cells and organisms. Science.

[2] Avidor-Reiss T., Gopalakrishnan J. (2013). Building a centriole. Curr. Opin. Cell Biol..

[3] Avidor-Reiss T., Khire A., Fishman E.L., Jo K.H. (2015). Atypical centrioles during sexual reproduction. Front. Cell Dev. Biol.

[4] Avidor-Reiss T. (2018). Rapid Evolution of Sperm Produces Diverse Centriole Structures that Reveal the Most Rudimentary Structure Needed for Function. Cells.

[5] Fishman E.L., Jo K., Ha A., Royfman R., Zinn A., Krishnamurthy M., Avidor-Reiss T. (2017). Atypical centrioles are present in Tribolium sperm. Open Biol..

[6] Khire A., Jo K.H., Kong D., Akhshi T., Blachon S., Cekic A.R., Hynek S., Ha A., Loncarek J., Mennella V. (2016). Centriole Remodeling during Spermiogenesis in Drosophila. Curr. Biol..

[7] Fishman E.L., Jo K., Nguyen Q.P.H., Kong D., Royfman R., Cekic A.R., Khanal S., Miller A.L., Simerly C., Schatten G. (2018). A novel atypical sperm centriole is functional during human fertilization. Nat. Commun..

[8] Gottardo M., Callaini G., Riparbelli M.G. (2015). Structural characterization of procentrioles in Drosophila spermatids. Cytoskeleton.

[9] Blachon S., Khire A., Avidor-Reiss T. (2014). The origin of the second centriole in the zygote of *Drosophila melanogaster*. Genetics.

[10] Avidor-Reiss T., Fishman E.L. (2018). It Takes Two (Centrioles) to Tango. Reproduction.

[11] Fu J., Lipinszki Z., Rangone H., Min M., Mykura C., Chao-Chu J., Schneider S., Dzhindzhev N.S., Gottardo M., Riparbelli M.G. (2016). Conserved molecular interactions in centriole-to-centrosome conversion. Nat. Cell Biol..

[12] Bettencourt-Dias M., Rodrigues-Martins A., Carpenter L., Riparbelli M., Lehmann L., Gatt M.K., Carmo N., Balloux F., Callaini G., Glover D.M. (2005). SAK/PLK4 is required for centriole duplication and flagella development. Curr. Biol..

[13] Blachon S., Gopalakrishnan J., Omori Y., Polyanovsky A., Church A., Nicastro D., Malicki J., Avidor-Reiss T. (2008). Drosophila asterless and vertebrate Cep152 Are orthologs essential for centriole duplication. Genetics.

[14] O’Connell K.F., Maxwell K.N., White J.G. (2000). The spd-2 gene is required for polarization of the anteroposterior axis and formation of the sperm asters in the Caenorhabditis elegans zygote. Dev. Biol..

[15] Delattre M., Leidel S., Wani K., Baumer K., Bamat J., Schnabel H., Feichtinger R., Schnabel R., Gonczy P. (2004). Centriolar SAS-5 is required for centrosome duplication in C. elegans. Nat. Cell Biol..

[16] Leidel S., Delattre M., Cerutti L., Baumer K., Gonczy P. (2005). SAS-6 defines a protein family required for centrosome duplication in C. elegans and in human cells. Nat. Cell Biol..

[17] Matsuura K., Lefebvre P.A., Kamiya R., Hirono M. (2004). Bld10p, a novel protein essential for basal body assembly in Chlamydomonas: Localization to the cartwheel, the first ninefold symmetrical structure appearing during assembly. J. Cell Biol..

[18] Hung L.Y., Tang C.J., Tang T.K. (2000). Protein 4.1 R-135 interacts with a novel centrosomal protein (CPAP) which is associated with the gamma-tubulin complex. Mol. Cell Biol..

[19] Goshima G., Wollman R., Goodwin S.S., Zhang N., Scholey J.M., Vale R.D., Stuurman N. (2007). Genes required for mitotic spindle assembly in Drosophila S2 cells. Science.

[20] Izquierdo D., Wang W.J., Uryu K., Tsou M.F. (2014). Stabilization of Cartwheel-less Centrioles for Duplication Requires CEP295-Mediated Centriole-to-Centrosome Conversion. Cell Reports.

[21] Chang C.W., Hsu W.B., Tsai J.J., Tang C.J., Tang T.K. (2016). CEP295 interacts with microtubules and is required for centriole elongation. J. Cell Sci..

[22] Megraw T.L., Li K., Kao L.R., Kaufman T.C. (1999). The centrosomin protein is required for centrosome assembly and function during cleavage in Drosophila. Development.

[23] Doxsey S.J., Stein P., Evans L., Calarco P.D., Kirschner M. (1994). Pericentrin, a highly conserved centrosome protein involved in microtubule organization. Cell.

[24] Bonaccorsi S., Giansanti M.G., Gatti M. (1998). Spindle self-organization and cytokinesis during male meiosis in asterless mutants of *Drosophila melanogaster*. J. Cell Biol..

[25] Blachon S., Cai X., Roberts K.A., Yang K., Polyanovsky A., Church A., Avidor-Reiss T. (2009). A proximal centriole-like structure is present in Drosophila spermatids and can serve as a model to study centriole duplication. Genetics.

[26] Avidor-Reiss T., Gopalakrishnan J., Blachon S., Polyanovsky A., Schatten H. (2012). Centriole duplication and inheritance in *Drosophila melanogaster*. The Centrosome: Cell and Molecular Mechanisms of Functions and Dysfunctions in Disease.

[27] Mottier-Pavie V., Megraw T.L. (2009). Drosophila bld10 is a centriolar protein that regulates centriole, basal body, and motile cilium assembly. Mol. Biol. Cell.

[28] Pearson C.G., Osborn D.P., Giddings T.H., Beales P.L., Winey M. (2009). Basal body stability and ciliogenesis requires the conserved component Poc1. J. Cell Biol..

[29] Keller L.C., Geimer S., Romijn E., Yates J., Zamora I., Marshall W.F. (2009). Molecular architecture of the centriole proteome: The conserved WD40 domain protein POC1 is required for centriole duplication and length control. Mol. Biol Cell.

[30] Venoux M., Tait X., Hames R.S., Straatman K.R., Woodland H.R., Fry A.M. (2013). Poc1A and Poc1B act together in human cells to ensure centriole integrity. J. Cell Sci..

[31] Hames R.S., Hames R., Prosser S.L., Euteneuer U., Lopes C.A., Moore W., Woodland H.R., Fry A.M. (2008). Pix1 and Pix2 are novel WD40 microtubule-associated proteins that colocalize with mitochondria in Xenopus germ plasm and centrosomes in human cells. Exp. Cell Res..

[32] Sarig O., Nahum S., Rapaport D., Ishida-Yamamoto A., Fuchs-Telem D., Qiaoli L., Cohen-Katsenelson K., Spiegel R., Nousbeck J., Israeli S. (2012). Short stature, onychodysplasia, facial dysmorphism, and hypotrichosis syndrome is caused by a POC1A mutation. Am. J. Hum. Genet..

[33] Shaheen R., Faqeih E., Shamseldin H.E., Noche R.R., Sunker A., Alshammari M.J., Al-Sheddi T., Adly N., Al-Dosari M.S., Megason S.G. (2012). POC1A truncation mutation causes a ciliopathy in humans characterized by primordial dwarfism. Am. J. Hum. Genet..

[34] Zhang C., Zhang Q., Wang F., Liu Q. (2015). Knockdown of poc1b causes abnormal photoreceptor sensory cilium and vision impairment in zebrafish. Biochem. Biophys. Res. Commun..

[35] Roosing S., Lamers I.J., de Vrieze E., van den Born L.I., Lambertus S., Arts H.H., Group P.B.S., Peters T.A., Hoyng C.B., Kremer H. (2014). Disruption of the basal body protein POC1B results in autosomal-recessive cone-rod dystrophy. Am. J. Hum. Genet..

[36] Sydor A.M., Coyaud E., Rovelli C., Laurent E., Liu H., Raught B., Mennella V. (2018). PPP1R35 is a novel centrosomal protein that regulates centriole length in concert with the microcephaly protein RTTN. eLife.

[37] Stevens N.R., Roque H., Raff J.W. (2010). DSas-6 and Ana2 coassemble into tubules to promote centriole duplication and engagement. Dev. Cell.

[38] Shields A.R., Spence A.C., Yamashita Y.M., Davies E.L., Fuller M.T. (2014). The actin-binding protein profilin is required for germline stem cell maintenance and germ cell enclosure by somatic cyst cells. Development.

[39] Wang C., Li S., Januschke J., Rossi F., Izumi Y., Garcia-Alvarez G., Gwee S.S., Soon S.B., Sidhu H.K., Yu F. (2011). An ana2/ctp/mud complex regulates spindle orientation in Drosophila neuroblasts. Dev. Cell.

[40] Cottee M.A., Muschalik N., Johnson S., Leveson J., Raff J.W., Lea S.M. (2015). The homo-oligomerisation of both Sas-6 and Ana2 is required for efficient centriole assembly in flies. eLife.

[41] Gopalakrishnan J., Guichard P., Smith A.H., Schwarz H., Agard D.A., Marco S., Avidor-Reiss T. (2010). Self-assembling SAS-6 multimer is a core centriole building block. J. Biol. Chem..

[42] Basiri M.L., Blachon S., Chim Y.C., Avidor-Reiss T. (2013). Imaging centrosomes in fly testes. J. Vis. Exp..

[43] Tates A.D. (1971). Cytodifferentiation during Spermatogenesis in Drosophila melanogaster: An. Electron. Microscope Study.

[44] Demarco R.S., Eikenes A.H., Haglund K., Jones D.L. (2014). Investigating spermatogenesis in *Drosophila melanogaster*. Methods.

[45] Chen D., McKearin D.M. (2003). A discrete transcriptional silencer in the bam gene determines asymmetric division of the Drosophila germline stem cell. Development.

[46] Stevens N.R., Dobbelaere J., Brunk K., Franz A., Raff J.W. (2010). Drosophila Ana2 is a conserved centriole duplication factor. J. Cell Biol.

[47] Cizmecioglu O., Arnold M., Bahtz R., Settele F., Ehret L., Haselmann-Weiss U., Antony C., Hoffmann I. (2010). Cep152 acts as a scaffold for recruitment of Plk4 and CPAP to the centrosome. J. Cell Biol.

[48] Guderian G., Westendorf J., Uldschmid A., Nigg E.A. (2010). Plk4 trans-autophosphorylation regulates centriole number by controlling betaTrCP-mediated degradation. J. Cell Sci.

[49] Hatch E.M., Kulukian A., Holland A.J., Cleveland D.W., Stearns T. (2010). Cep152 interacts with Plk4 and is required for centriole duplication. J. Cell Biol..

[50] Habedanck R., Stierhof Y.D., Wilkinson C.J., Nigg E.A. (2005). The Polo kinase Plk4 functions in centriole duplication. Nat. Cell Biol..

[51] Gottardo M., Callaini G., Riparbelli M.G. (2014). Procentriole assembly without centriole disengagement-a paradox of male gametogenesis. J. Cell Sci..

[52] Dzhindzhev N.S., Tzolovsky G., Lipinszki Z., Schneider S., Lattao R., Fu J., Debski J., Dadlez M., Glover D.M. (2014). Plk4 phosphorylates Ana2 to trigger Sas6 recruitment and procentriole formation. Curr. Biol..

[53] Saurya S., Roque H., Novak Z.A., Wainman A., Aydogan M.G., Volanakis A., Sieber B., Pinto D.M., Raff J.W. (2016). Drosophila Ana1 is required for centrosome assembly and centriole elongation. J. Cell Sci..

[54] Avidor-Reiss T., Turner K., Kloc M. The Evolution of Centriole Structure: Heterochrony, Neoteny, and Hypermorphosis. Centriole and Golgi.

[55] Nakazawa Y., Hiraki M., Kamiya R., Hirono M. (2007). SAS-6 is a cartwheel protein that establishes the 9-fold symmetry of the centriole. Curr. Biol..

[56] Guichard P., Hamel V., Gonczy P. (2018). The Rise of the Cartwheel: Seeding the Centriole Organelle. Bioessays.

[57] Li S., Fernandez J.J., Marshall W.F., Agard D.A. (2019). Electron cryo-tomography provides insight into procentriole architecture and assembly mechanism. eLife.

[58] Bayless B.A., Galati D.F., Junker A.D., Backer C.B., Gaertig J., Pearson C.G. (2016). Asymmetrically localized proteins stabilize basal bodies against ciliary beating forces. J. Cell Biol.

[59] Bayless B.A., Giddings T.H., Winey M., Pearson C.G. (2012). Bld10/Cep135 stabilizes basal bodies to resist cilia-generated forces. Mol. Biol Cell.

