# Supplementary material for: Poc1B and Sas-6 Function Together during the Atypical Centriole Formation in Drosophila melanogaster
Source: Cells. 2019 Aug 5;8(8):841. doi: 10.3390/cells8080841 (PMC6721650; doi:10.3390/cells8080841)
Supplement: Supplementary file 1 [file cells-08-00841-s001.pdf]

Supplementary figures

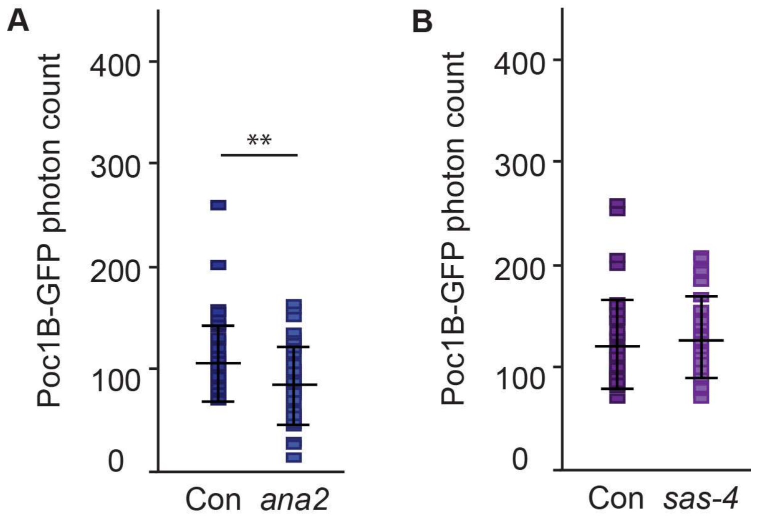

Supplemental Figure 1: Poc1B recruitment is reduced in ana2 but not in sas-4 heterozygote mutants.

No significant difference in gPoc1BGFP level in the PCL was observed in control (Con, sibling controls) (N= 47) compared to *ana2* (N= 36) (**A**) or *sas-4* (N= 47) (**B**) heterozygote mutant background. All images and quantification data were obtained by live cell imaging. **, P≤0.01

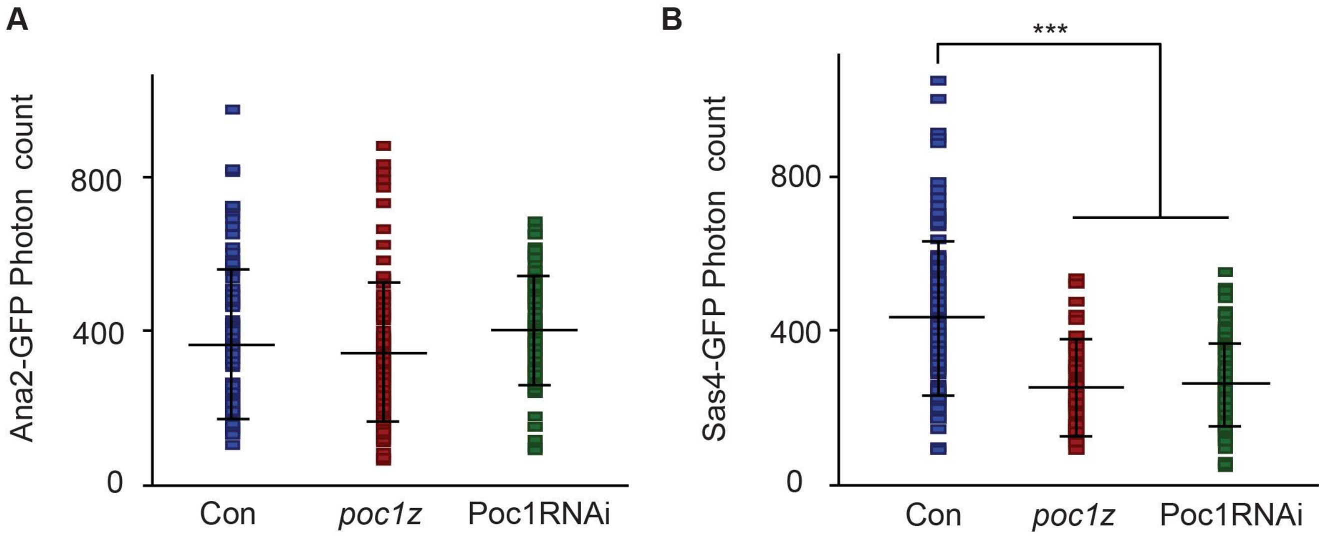

Supplemental Figure 2: Ana2 is not affected and Sas-4 is reduced in poc1 mutant and knockdown

Ana2-GFP localization to the PCL was not affected in *poc1* loss of function mutant (*poc1*^{W87X}) (N= 85) and Poc1RNAi expressing flies (N= 70) compared to the control flies (N= 87) (**A**), but Sas-4GFP localization to the PCL was significantly reduced in *poc1* loss of function mutant (*poc1*^{W87X}) (N= 94) and Poc1RNAi expressing flies (N= 97) compared to the control flies (N= 99) (**B**). All images and quantification data were obtained by live cell imaging. ***, $P<0.001$

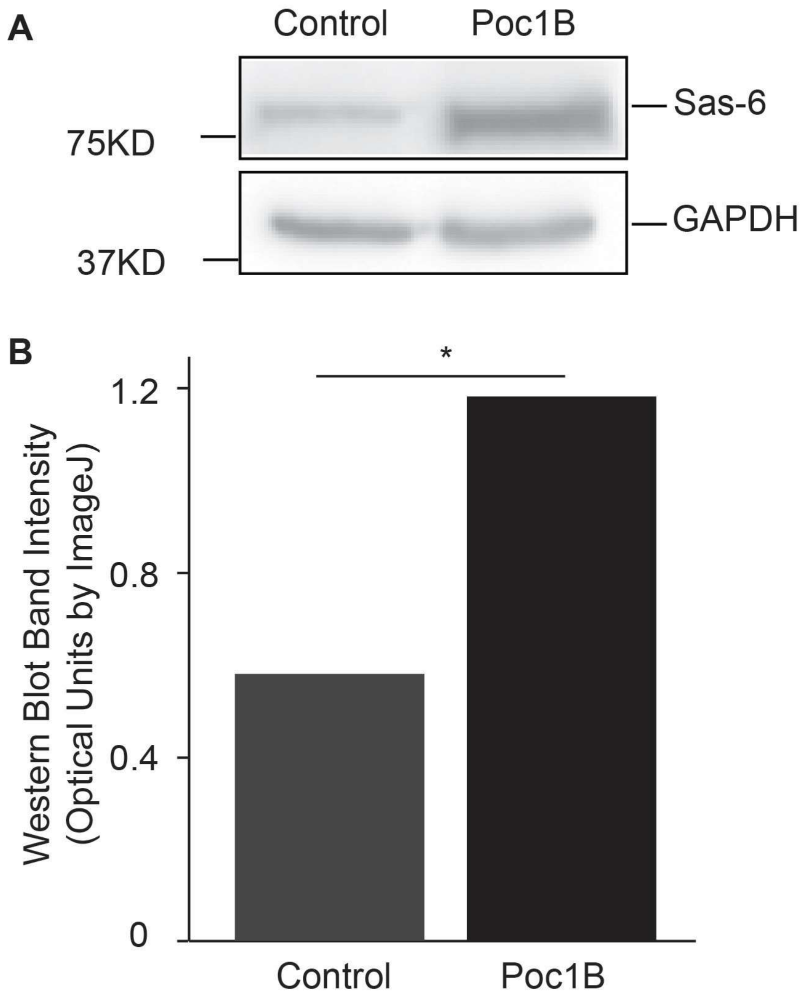

Supplemental Figure 3: Sas-6-GFP expression increases in Poc1B overexpressing flies.

A) A western blot using Sas-6 antibody show significant increase of Sas-6-GFP expression in the testis protein extracts of Poc1B overexpressing flies compared to the control flies.

B) The western blot experiments were repeated 6 times and showed significant increase of Sas-6-GFP expression (P=0.02). *, P<0.05

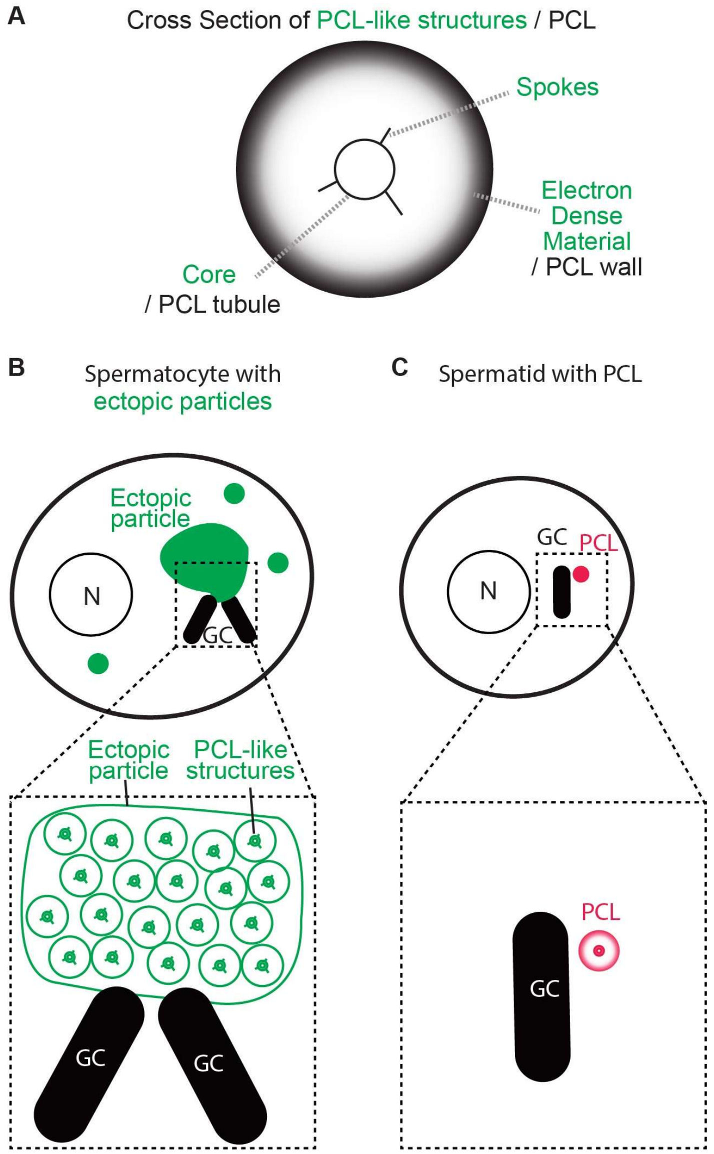

Supplemental Figure 4: Ectopic particles contain PCL-like structures.

A) Diagram representing the structural similarity between ectopic particles and PCL. Both are composed of a tubule-like structure in the center surrounded by electron-dense material. Sometimes spikes connected to the central tubule are observed.

B) Diagram representing the ectopic particles composed of PCL-like structures in a spermatocyte relative to the GC.

C) Diagram representing the PCL in a spermatid relative to the GC.

GC, Giant Centriole; PCL, Proximal Centriole-Like structure.

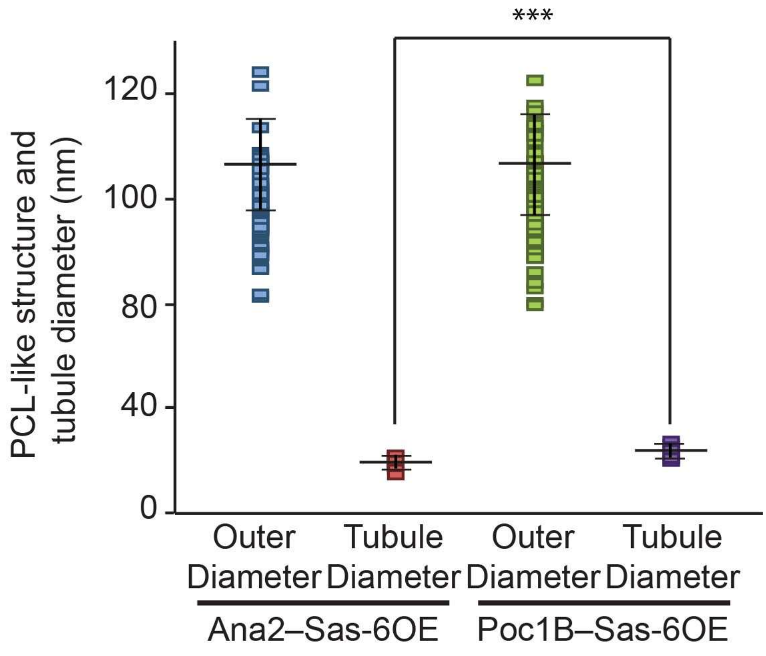

Supplemental Figure 5: PCL-like structure outer and tubule diameters

A) In mature spermatocytes, the outer diameters of the PCL-like structures were similar in Ana2GFP–Sas-6GFP co-overexpressed (N =123) and Poc1BGFP–Sas-6GFP co-overexpressed flies (N =90), but the tubule diameters were significantly wider in Poc1B–Sas-6 co-overexpressed flies (N =16) compared to Ana2GFP–Sas-6GFP co-overexpressing flies (N =17). OE, overexpression/co-overexpression, ***, $P<0.001$.

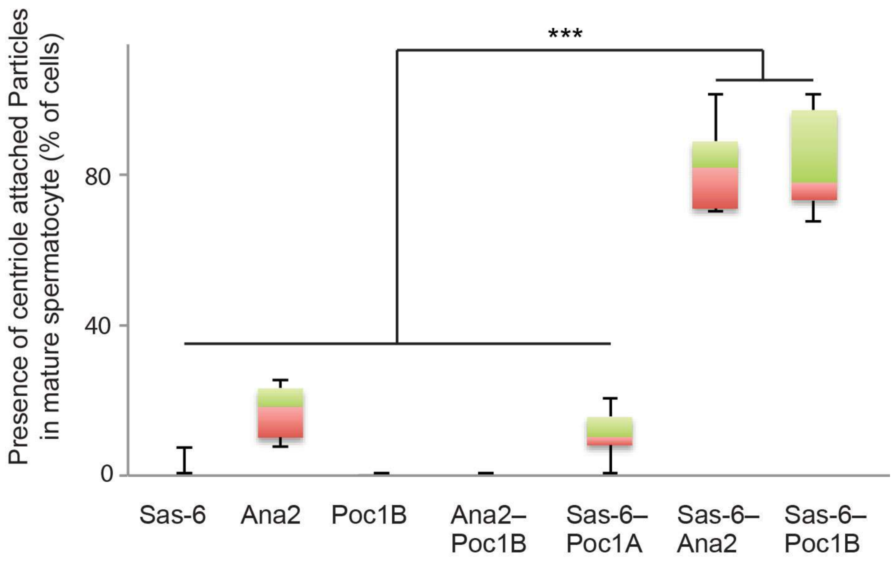

Supplemental Figure 6: Sas-6-Ana2 and Sas-6-Poc1B specifically induce the formation of centriole-attached particles.

The percentages of centriole-attached particles in mature spermatocyte represented in box and whisker plot. Overexpression of Sas-6, Ana2, or Poc1B alone did not induce the formation of the particles. The co-overexpression of Ana2–Poc1B and Sas-6–Poc1A did not induce the formation of the particles, but Sas-6–Ana2 or Sas-6–Poc1B induced the formation of a large number of the centriole-attached particles in mature primary spermatocytes. N =10, ***, P<0.001
